# The Association of Drug-Funding Reimbursement With Survival Outcomes and Use of New Systemic Therapies Among Patients With Advanced Pancreatic Cancer

**DOI:** 10.1001/jamanetworkopen.2021.33388

**Published:** 2021-11-15

**Authors:** Michael J. Raphael, William Raskin, Steven Habbous, Xiaochen Tai, Jaclyn Beca, Wei F. Dai, Jessica Arias, Leta Forbes, Scott Gavura, James J. Biagi, Craig C. Earle, Kelvin K. W. Chan

**Affiliations:** 1 Division of Medical Oncology, Department of Medicine, Sunnybrook Health Sciences Centre, University of Toronto, Toronto, Ontario, Canada; 2 Canadian Centre for Applied Research in Cancer Control, Toronto, Ontario, Canada; 3 Provincial Drug Reimbursement Program, Ontario Health (Cancer Care Ontario), Toronto, Ontario, Canada; 4 Department of Oncology, William Osler Health System, Brampton, Ontario, Canada; 5 Department of Oncology, Queen’s University, Kingston, Ontario, Canada

## Abstract

**Importance:**

Gemcitabine-nab-paclitaxel (GEMNAB) and fluorouracil, leucovorin, irinotecan, and oxaliplatin (FOLFIRINOX) both improve survival of patients with advanced pancreatic cancer when compared with single-agent gemcitabine in clinical trials.

**Objective:**

To describe changes in the survival of patients with advanced pancreatic cancer associated with sequential drug-funding approvals and to determine if there exist distinct patient populations for whom GEMNAB and FOLFIRINOX are associated with survival benefit.

**Design, Setting, and Participants:**

This population-based, retrospective cohort study examined all incident cases of advanced pancreatic cancer treated with first-line chemotherapy in Ontario, Canada (2008-2018) that were identified from the Cancer Care Ontario (Ontario Health) New Drug Funding Program database. Statistical analysis was performed from October 2020 to January 2021.

**Exposures:**

First-line chemotherapy for advanced pancreatic cancer.

**Main Outcomes and Measures:**

The main outcomes were the proportion of patients treated with each chemotherapy regimen over time and overall survival for each regimen. Cox proportional hazards regression models were used to compare overall survival between treatment regimens after adjustment for confounding variables, inverse probability of treatment weighting, and matching.

**Results:**

From 2008 to 2018, 5465 patients with advanced pancreatic cancer were treated with first-line chemotherapy in Ontario, Canada. The median (range) age of patients was 66.9 (27.8-93.4) years; 2447 (45%) were female; 878 (16%) had prior pancreatic resection, and 328 (6%) had prior adjuvant gemcitabine. During the time period when only gemcitabine and FOLFIRINOX were funded (2011-2015), 49% (929 of 1887) received FOLFIRINOX. When GEMNAB was subsequently funded (2015-2018), 9% (206 of 2347) received gemcitabine, 44% (1034 of 2347) received FOLFIRINOX, and 47% (1107 of 2347) received GEMNAB. The median overall survival increased from 5.6 months (95% CI, 5.1-6.0 months) in 2008 to 2011 to 6.9 months (95% CI, 6.5-7.4 months) in 2011 to 2015 to 7.6 months (95% CI, 7.1-8.0 months) in 2015 to 2018. Patients receiving FOLFIRINOX were younger and healthier than patients receiving GEMNAB. After adjustment and weighting, FOLFIRINOX was associated with better overall survival than GEMNAB (hazard ratio [HR], 0.75 [95% CI, 0.69-0.81]). In analyses comparing patients treated with GEMNAB and gemcitabine, GEMNAB was associated with better overall survival (HR, 0.86 [95% CI, 0.78-0.94]).

**Conclusions and Relevance:**

This cohort study of patients with advanced pancreatic cancer receiving first-line palliative chemotherapy within a universal health care system found that drug funding decisions were associated with increased uptake of new treatment options over time and improved survival. Both FOLFIRINOX and GEMNAB were associated with survival benefits in distinct patient populations.

## Key Points

**Question:**

Are drug-funding reimbursement decisions associated with uptake of new therapies and survival outcomes for patients with advanced pancreatic cancer?

**Findings:**

In this population-based cohort study of 5465 patients with advanced pancreatic cancer receiving first-line chemotherapy within a universal health care system, reimbursement decisions were associated with uptake of new treatment options over time. Uptake of both FOLFIRINOX and gemcitabine combined with nab-paclitaxel were associated with improvements in survival for patients with pancreatic cancer.

**Meaning:**

These findings suggest that drug-funding reimbursement decisions are associated with uptake of new cancer therapies and improvements in survival for patients with advanced pancreatic cancer.

## Teaser

This cohort study investigates whether there is an association of sequential drug-funding approvals and overall survival of patients with advanced pancreatic cancer.

## Introduction

The cornerstone of treatment for metastatic pancreatic cancer is palliative chemotherapy. Based on evidence from 3 randomized clinical trials, single-agent gemcitabine,^1^ gemcitabine and nab-paclitaxel (GEMNAB),^2^ and fluorouracil, leucovorin, irinotecan, and oxaliplatin (FOLFIRINOX)^3^ are the 3 most commonly used first-line palliative chemotherapy regimens.

The 3 randomized trials that demonstrated the efficacy of these agents all enrolled different patient populations. The trial evaluating gemcitabine monotherapy enrolled patients with symptomatic, locally advanced or metastatic pancreatic cancer, up to age 79 years, with a Karnofsky performance status (KPS) of 50 to 80.^1^ The trial evaluating GEMNAB included patients with metastatic pancreatic cancer, up to age 88 years, with a KPS of 70 or higher. The trial evaluating FOLFIRINOX included patients with metastatic pancreatic cancer, up to age 76 years, who had an Eastern Cooperative Oncology Group (ECOG) performance status of 0 to 1 (approximately equal to a KPS of 90 to 100).^4^

Compared with fluorouracil, single-agent gemcitabine was shown to improve the clinical benefit response, a composite end point of pain control, performance status, and weight maintenance.^1^ Compared with single-agent gemcitabine, GEMNAB, and FOLFIRINOX improved median overall survival by 1.8 months (8.5 vs 6.7 months) and 4.3 months (11.1 vs 6.8 months), respectively.^2,3^ FOLFIRINOX was also shown to improve quality of life compared with single-agent gemcitabine, whereas no quality of life information was reported from the GEMNAB trial.^5^

These 3 clinical trials show that GEMNAB and FOLFIRINOX result in better overall survival than single-agent gemcitabine and are thus more suitable treatment options for patients with good performance status.^6^ However, these trials leave unanswered whether GEMNAB and FOLFIRINOX can be used interchangeably or whether they benefit distinct subsets of patients. This is an important question for health system payers and policy makers when making drug reimbursement decisions. Nab-paclitaxel is an expensive medication and previous economic analyses from both Canada^7^ and the United Kingdom^8^ have shown FOLFIRINOX to be a more cost-effective regimen than GEMNAB. Moreover, there is a considerable difference in median overall survival when GEMNAB is used in the clinical trial setting compared with when it is used in routine clinical practice (median 8.5 months vs 6.5 months, respectively).^9^ Thus, the modest survival benefits observed with GEMNAB over gemcitabine in the clinical trial setting may not translate into survival benefits when these medications are used in routine clinical practice.^10,11^

In the Canadian province of Ontario, there is a publicly funded, universal cancer care system that provides reimbursement for new injectable cancer medicines. Prior to November 2011, gemcitabine was the only funded first-line treatment for advanced pancreatic cancer. FOLFIRINOX was funded for treatment of metastatic pancreatic cancer in November 2011 and expanded to use in locally advanced and unresectable disease in April 2015. GEMNAB was funded for use in locally advanced and metastatic pancreatic cancer in April 2015. The serial approval and universal funding of these agents provides a unique opportunity to examine trends in the selection of first-line chemotherapy for advanced pancreatic cancer and whether these sequential drug approvals are associated with population-level survival benefits for distinct subsets of patients.

Therefore, the main objective of this study was to describe changes in the survival of patients with advanced pancreatic cancer associated with sequential drug funding approvals and to determine if there exist patient populations for whom GEMNAB and FOLFIRINOX are associated with survival benefit .

## Methods

### Study Design

This population-based, retrospective cohort study examined patients with locally advanced and unresectable or metastatic pancreatic cancer in Ontario, Canada. This study was approved by the Research Ethics Board of Sunnybrook Health Sciences Centre and was designed, analyzed, and reported in accordance with the Strengthening the Reporting of Observational Studies in Epidemiology (STROBE) reporting guideline.^16^ The need for informed consent was waived because all data were routinely collected administrative data.

### Study Population, Data Sources, and Definitions

Patients were eligible for inclusion in this study if they received at least 1 dose of first-line gemcitabine, GEMNAB, or FOLFIRINOX for locally advanced and unresectable or metastatic pancreas cancer between November 7, 2008, and December 31, 2018, in Ontario, Canada. Patients were identified using the New Drug Funding Program (NDFP) database. Once an injectable cancer medicine has been approved for funding coverage in Ontario, the NDFP pays for all costs of the drug. To obtain funding, clinicians must enroll their patient in the NDFP and provide information to support that the patient meets the drug funding eligibility criteria. Thus, the NDFP database is a comprehensive resource containing demographic and clinical factors for all incident cases of pancreatic cancer in Ontario that are treated with first-line chemotherapy. Race and ethnicity data were not collected in this study, as this information is not available in the population-level administrative databases.

Cases identified in the NDFP databases were deterministically linked by using their Ontario provincial health insurance number to administrative health care databases housed at Ontario Health (Cancer Care Ontario) to ascertain sociodemographic information and information on previous pancreatic resection or radiation, baseline health care use, and overall survival. A complete description of the health care administrative databases can be found in eTable 1 in the Supplement.

Comorbidity was classified using the modified Charlson Comorbidity Index, which was based on all noncancer diagnoses recorded during any hospital admission in the 2 years before their diagnosis date.^12^ Median neighborhood income quintile, immigrant density, and rurality were obtained from the 2006 Canada Census based on the patients’ residential postal code at the time they were enrolled in the NDFP policy or their postal code at the time of diagnosis.^13^

### Statistical Analysis

Data are presented as counts, proportions, and means with corresponding standard deviations. Comparisons of proportions were made using the χ^2^ test. To estimate the association of patient, physician, and hospital factors with chemotherapy regimen selection, a series of logistic regression models were constructed. Associations are reported as odds ratios (OR) with 95% CIs.

First, to estimate the association between chemotherapy regimen selection and overall survival in the most recent period when both FOLFIRINOX and GEMNAB were funded (2015-2018), we used multivariable Cox proportional hazards regression, presenting adjusted hazard ratios (aHR) with 95% CI. Patients were censored after 2 years of follow-up or December 31, 2020, whichever came first. Survival analyses were weighted by the inverse probability of treatment (IPT).^14,15^ Weights were estimated using a logistic regression model with treatment group set as the dependent variable and then regressed on potentially confounding variables, including age, sex, rural-urban status, income quintile, comorbidity score, baseline emergency department visits and hospitalizations, immigrant population density, ECOG performance status, previous history of pancreatic resection, and extent of pancreatic cancer (unresectable locally advanced vs metastatic). These variables were selected a priori based on clinical knowledge of the factors that might affect treatment assignment and independently affect outcome. The weighted standardized difference was calculated for all the baseline characteristics to assess the balance of the baseline characteristics in the cohort after applying the weight. A standard difference of less than 0.1 is interpreted to indicate appropriate balance between groups.^14,16^ An unweighted adjusted model was also performed, using the same aforementioned factors as covariates.

Second, in order to evaluate whether GEMNAB was associated with benefit for a population of patients distinct from FOLFIRINOX, we compared the survival outcomes of patients with GEMNAB in the most recent period (2015-2018) with those patients treated with gemcitabine in the preceding period (2011-2015) when only gemcitabine and FOLFIRINOX were funded and GEMNAB was not. We performed 3 Cox proportional hazards regression models. The first was weighted by the IPT, although the IPT weight did not include ECOG since this was unavailable for the gemcitabine-alone group. The second was a multivariable Cox proportional hazards regression model adjusted for all aforementioned sociodemographic and clinical variables. In the third model, patients were hard matched on sex (male vs female), comorbidity score (0 vs ≥1), baseline emergency department visit use (0 vs ≥1), previous adjuvant chemotherapy (yes vs no), and previous pancreatic surgery (yes vs no) and matched to the nearest neighbor on age at first treatment using the *MatchIt* package in R.^17^ Stratified Cox proportional hazards regression (stratified by the matching identifier) was used to accommodate the matching.

All statistical analyses were conducted using SAS version 9.4 (SAS Institute) and R software version 4.0.3 (R Project for Statistical Computing) from October 2020 to January 2021. *P* < .05 were considered statistically significant, but an evaluation of the effect size and CIs should be used to evaluate the clinical significance of results.

## Results

### Patient Characteristics

Between November 7, 2008, and December 31, 2018, 5465 patients were diagnosed with advanced pancreatic adenocarcinoma and received at least 1 dose of first-line palliative chemotherapy. The median (range) age of patients was 66.9 (27.8-93.4) years; 2447 (45%) were female, and 4777 (87%) were urban-dwelling. Prior pancreatic resection was performed in 878 patients (16%) and 328 (6%) had previous adjuvant single-agent gemcitabine chemotherapy (Table 1).

**Table 1.** Baseline Characteristics of the Patient Populations Treated With First-line Gemcitabine, FOLFIRINOX, or Gemcitabine Plus Nab-paclitaxel in Ontario, Canada^a^

Characteristic	No. (%)					
	Gemcitabine			FOLFIRINOX		Gemcitabine/nab-paclitaxel
	2008-2011 (n = 1231)^a^	2011-2015 (n = 958)^a^	2015-2018 (n = 206)^a^	2011-2015 (n = 929)^a^	2015-2018 (n = 1034)^a^	2015-2018 (n = 1107)^a^
Total follow-up time, d	170 (79-354)	162 (66-355)	136 (51-252)	252 (128-455)	235 (109-425)	164 (74-289)
Patient sociodemographic characteristics						
Age, mean (SD), y	66 (10.5)	70 (9.3)	76 (9.5)	62 (9.1)	62 (8.9)	70 (8.8)
Sex						
Female	596 (48)	434 (45)	103 (50)	396 (43)	456 (44)	462 (42)
Male	635 (52)	524 (55)	103 (50)	533 (57)	578 (56)	645 (58)
Rurality						
Urban	1080 (88)	850 (89)	176 (85)	783 (84)	910 (88)	978 (88)
Rural	150 (12)	108 (11)	30 (15)	146 (16)	124 (12)	129 (12)
Income quintile						
Highest	247 (20)	223 (23)	51 (25)	246 (27)	249 (24)	229 (21)
Mid-high	244 (20)	219 (23)	49 (24)	221 (24)	240 (23)	248 (22)
Middle	260 (21)	196 (20)	38 (19)	150 (16)	210 (20)	242 (22)
Mid-low	262 (21)	168 (18)	36 (18)	164 (18)	197 (19)	214 (19)
Lowest	211 (17)	152 (16)	31 (15)	147 (16)	135 (13)	170 (15)
Immigrant density						
Least	713 (59)	516 (54)	118 (58)	585 (63)	631 (61)	683 (62)
Mid	300 (25)	263 (28)	43 (21)	210 (23)	255 (25)	256 (23)
Most	205 (17)	174 (18)	44 (21)	128 (14)	141 (14)	156 (14)
Clinical characteristics						
Charlson comorbidity score						
0	953 (77)	692 (72)	143 (69)	741 (80)	798 (77)	828 (75)
1	196 (16)	198 (21)	40 (19)	152 (16)	180 (17)	204 (18)
≥2	82 (7)	68 (7)	23 (11)	36 (4)	56 (5)	75 (7)
ECOG status^b^						
0	NA	NA	28 (14)	NA	404 (39)	207 (19)
≥1	NA	NA	175 (86)	NA	623 (61)^c^	898 (81)
Disease extent^d^						
Locally advanced	NA	NA	62 (31)	0 (0)	365 (36)	290 (26)
Metastatic	NA	NA	141 (69)	929 (100)	663 (65)	815 (74)
No. of emergency department visits 6 mo within chemotherapy start date						
0	524 (43)	401 (42)	89 (43)	523 (56)	565 (55)	578 (52)
1	230 (19)	208 (22)	36 (17)	130 (14)	156 (15)	157 (14)
≥2	477 (39)	349 (36)	81 (39)	276 (30)	313 (30)	372 (34)
No. of hospital admissions visits 6 mo within chemotherapy start date						
0	438 (36)	348 (36)	84 (41)	487 (52)	541 (52)	542 (49)
1	471 (38)	355 (37)	63 (31)	268 (29)	318 (31)	329 (30)
≥2	322 (26)	255 (27)	59 (29)	174 (19)	175 (17)	236 (21)
Prior pancreatic resection						
No	1005 (82)	791 (83)	172 (83)	780 (84)	875 (85)	964 (87)
Yes	226 (18)	167 (17)	34 (17)	149 (16)	159 (15)	143 (13)

Abbreviations: FOLFIRINOX, fluorouracil, leucovorin, irinotecan, and oxaliplatin; NA, not applicable.

^a^ Period 1 = November 7, 2008 to November 6, 2011; Period 2 = November 7, 2011 to April 14, 2015; Period 3 = April 15, 2015 to December 31, 2018.

^b^ Eastern Cooperative Oncology Group (ECOG) score submission became a requirement in the most recent period.

^c^ No patient receiving FOLFIRINOX in this period had ECOG score greater than 1

^d^ Disease extent was a requirement for all enrollment forms in the most recent period.

### Trends and Factors Associated With First-line Chemotherapy Selection

The proportion of patients treated with first-line single-agent gemcitabine decreased from 100% (1231 of 1231) in 2008 to 2011 to 51% (958 of 1887) in 2011 to 2015 to 9% (206 of 2347) 2015 to 2018. Over time, single-agent gemcitabine was used in a serially restricting cohort of patients who were older and more comorbid (Table 1). The median (SD) age of patients treated with gemcitabine increased from 66 (10.5) years in 2008 to 2011 to 70 (9.3) years in 2011 to 2015 to 76 (9.5) years in 2015 to 2018, whereas the proportion of patients with a comorbidity score of 0 or 1 decreased from 93% (1149 of 1231) to 47% (890 of 1887) to 8% (183 of 2347) in the same periods, respectively.

During the period when only gemcitabine and FOLFIRINOX were funded for treatment of pancreatic cancer, 51% (958 of 1887) received gemcitabine and 49% (929 of 1887) received FOLFIRINOX and it took nearly 1 year for FOLFIRINOX to become the most commonly selected first-line treatment option (Figure). When GEMNAB was funded, there was an immediate decline in the use of single-agent gemcitabine. When all 3 agents were funded for treatment of pancreatic cancer, 9% (206 of 2347) received gemcitabine, 44% (1034 of 2347) received FOLFIRINOX, and 47% (1107 of 2347) received GEMNAB.

**Figure.**
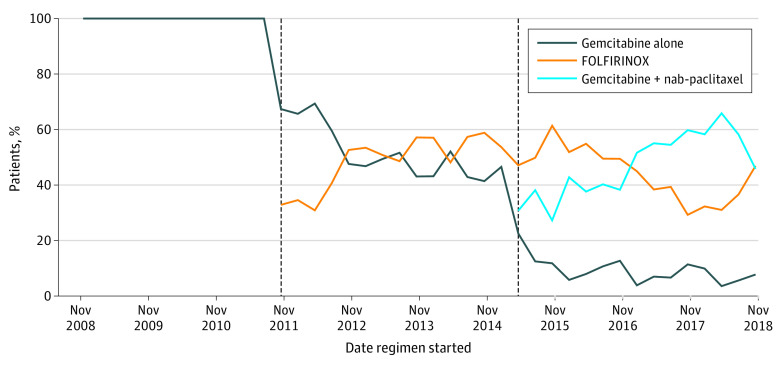
Proportion of Patients With Advanced Pancreatic Cancer Treated With First-line Single-Agent Gemcitabine, Gemcitabine and Nab-paclitaxel, and FOLFIRINOX Between November 2008 and December 2018

In the most recent period (2015-2018) in which both FOLFIRINOX and GEMNAB were funded, FOLFIRINOX was more likely to be selected for younger patients (adjusted odds ratio [aOR] per each additional year of age, 0.90; 95% CI, 0.89-0.92), more affluent patients (highest vs lowest income quintile: aOR, 1.84; 95% CI, 1.25-2.71), patients with better performance status (ECOG 0 vs ≥1: aOR, 2.65; 95% CI, 2.08-3.37), patients with less extent of disease (locally advanced vs metastatic: aOR, 2.01; 95% CI, 2.00; 1.58-2.56) and among those treated with prior gemcitabine in the adjuvant setting (aOR, 2.54; 95% CI, 1.57-4.09) (Table 2).

**Table 2.** Factors Associated With Selection of FOLFIRINOX (N = 1034) vs Gemcitabine and Nab-paclitaxel (N = 1107) as the First-line Treatment for Advanced Pancreatic Cancer Between April 2015 and December 2018

Characteristic	Crude		Adjusted^a^	
	OR (95% CI)	*P* value	OR (95% CI)	*P* value
Sociodemographic factors				
Age treatment started (per year)	0.91 (0.90-0.92)	<.001	0.90 (0.89-0.92)	<.001
Female vs male	1.10 (0.93-1.31)	.27	1.07 (0.86-1.32)	.56
Urban vs rural	0.97 (0.74-1.26)	.81	0.81 (0.56-1.14)	.23
Income quintile				
Lowest	1 [Reference]	.24	1 [Reference]	.03
Mid-low	1.16 (0.86-1.56)		1.68 (1.15-2.46)	
Middle	1.09 (0.82-1.46)		1.60 (1.09-2.34)	
Mid-high	1.22 (0.91-1.62)		1.50 (1.03-2.18)	
Highest	1.37 (1.03-1.83)		1.84 (1.25-2.71)	
Immigrant density				
Most dense	1 [Reference]	.73	1 [Reference]	.31
Mid-dense	1.10 (0.83-1.47)		0.75 (0.52-1.09)	
Least dense	1.02 (0.79-1.32)		0.83 (0.58-1.20)	
Clinical factors				
Charlson comorbidity				
0	1 [Reference]	.31	1 [Reference]	.61
1	0.92 (0.73-1.14)		1.11 (0.84-1.46)	
≥2	0.77 (0.54-1.11)		1.21 (0.75-1.98)	
ECOG (0 vs 1+)	2.81 (2.31-3.42)	<.001	2.65 (2.08-3.37)	<.001
uLAPC vs mPC	1.55 (1.29-1.86)	<.001	2.01 (1.58-2.56)	<.001
Previous treatment				
Gemcitabine	1.78 (1.33-2.39)	<.001	2.54 (1.57-4.09)	<.001
Radiation	0.77 (0.56-1.06)	.11	0.83 (0.54-1.26)	.38
Pancreatic resection	1.23 (0.96-1.56)	.10	1.04 (0.69-1.55)	.86
No. of baseline emergency department visits				
0	1 [Reference]	.25	1 [Reference]	.08
1	1.02 (0.79-1.31)		1.57 (0.78-3.15)	
≥2	0.86 (0.71-1.04)		1.06 (0.53-2.09)	
No. of baseline hospital admissions				
0	1 [Reference]	.03	1 [Reference]	.58
1	0.97 (0.80-1.18)		0.73 (0.38-1.41)	
≥2	0.74 (0.59-0.93)		0.68 (0.33-1.41)	
Topography				
Head of pancreas	1 [Reference]	.34	1 [Reference]	.74
Body of pancreas	0.98 (0.76-1.25)		1.01 (0.73-1.38)	
Tail of pancreas	1.10 (0.86-1.41)		1.19 (0.87-1.64)	
Physician and hospital factors				
International medical graduate^b^	1.60 (1.32-1.95)	<.001	1.95 (1.51-2.52)	<.001
Years since medical degree obtained	1.01 (1.00-1.01)	.13	1.00 (0.99-1.01)	.53
Facility type				
Teaching hospital	1 [Reference]	.002	1 [Reference]	.92
Small or community hospital	0.76 (0.64-0.90)		0.98 (0.72-1.35)	
Hospital volume (per 100 patients)	1.08 (1.04-1.11)	<.001	1.00 (1.00-1.00)	.18

Abbreviations: ECOG, Eastern Cooperative Oncology Group; FOLFIRINOX, fluorouracil, leucovorin, irinotecan, and oxaliplatin; OR, odds ratio; uLAPC, unresectable and locally advanced pancreatic cancer.

^a^ Adjusted for all variables in table.

^b^ Medical training outside Canada.

### Temporal Trends in Survival Outcomes

The median (IQR) follow-up duration was 209 (94-406) days. The median overall survival increased from 5.6 months (95% CI, 5.1-6.0 months) in 2008 to 2011 to 6.9 months (95% CI, 6.4-7.4 months) in 2011 to 2015 to 7.7 months (95% CI, 7.3-8.2 months) in 2015 to 2018 (eFigure 1 in the Supplement). Patients treated with FOLFIRINOX in the most recent period had the best overall survival (eFigure 2 in the Supplement). In the most recent period the crude median overall survival was 5.3 months (95% CI, 4.4-5.8 months) for gemcitabine, 6.7 months (95% CI, 6.2-7.2 months) for GEMNAB, and 10.1 months (95% CI, 9.4-10.9 months) for FOLFIRINOX. Mean time until censoring was 22.2 months for gemcitabine in 2008 to 2011, 22.8 months for gemcitabine in 2011 to 2015, and 23.0 months for FOLFIRINOX in 2011 to 2015. Mean time until censoring in 2015 to 2018 was 22.6 months for gemcitabine, 23.0 months for FOLFIRINOX, and 22.3 months for GEMNAB.

### Comparison of First-line FOLFIRINOX and GEMNAB

In unadjusted models, FOLFIRINOX was associated with better overall survival than GEMNAB (median overall survival, 10.1 months [95% CI, 9.4-10.9 months] vs 6.7 months [95% CI, 6.2-7.2 months]) (eFigure 3 and 4 in the Supplement). After IPT weighting, there was excellent balance between GEMNAB and FOLFIRINOX treatment groups with all standardized differences less than 0.1 (eTable 2 in the Supplement). FOLFIRINOX was associated with better overall survival compared with GEMNAB in adjusted models with IPT weighting (weighted aHR, 0.78; 95% CI, 0.73-0.83) and without IPT weighting (aHR 0.78; 95% CI, 0.71-0.87) (eTable 3 in the Supplement).

### Comparisons of First-Line Gemcitabine and GEMNAB

In the most recent period (2015-2018) in which all 3 first-line chemotherapy regimens were funded, in unadjusted models, GEMNAB was associated with a better overall survival than gemcitabine (median overall survival, 6.7 months [95% CI, 6.2-7.2 months] vs 5.3 months [95% CI, 4.4-5.8 months]). However, the treatment groups were so different in baseline characteristics that IPT weighted balance could not be achieved.

Patients treated with GEMNAB in the most recent period (2015-2018) had better overall survival than those treated with gemcitabine in the immediately preceding period (2011-2015) (aHR, 0.86 [95% CI, 0.78-0.94]; IPT weighted aHR, 0.86 [95% CI, 0.80-0.92]; and matched aHR, 0.78 [95% CI, 0.67-0.90]) (Table 3).

**Table 3.** Analyses of Overall Survival in Patients Treated With Gemcitabine + Nab-paclitaxel (2015-2018) Compared With Patients Treated With Gemcitabine (2011-2015)

Characteristic	Median overall survival	Crude Cox proportional model	Cox proportional hazard model		
			Adjusted^a^	Weighted	Matched^b^
Gemcitabine + nab-paclitaxel (n = 1107) vs gemcitabine (n = 958)	6.5 vs 5.3 mo	0.92 (0.84-1.01)	0.86 (0.78-0.94)	0.86 (0.80-0.92)	0.78 (0.67-0.90)
*P* value	.15	NA	NA	NA	NA

Abbreviations: GEMNAB, Gemcitabine-Nab-paclitaxel; NA, not applicable.

^a^ Adjusted for age at first treatment, sex, urban-rural status, income quintile, immigration density, Charlson Comorbidity Score, prior adjuvant chemotherapy, prior adjuvant radiation, prior resection, number of emergency department visits within 6 months of first dose of palliative chemotherapy, and number of hospital admissions within 6 months of first dose of palliative chemotherapy.

^b^ Matched on age at first treatment, sex, Charlson Comorbidity Score, prior adjuvant chemotherapy, prior resection, number of emergency department visits within 6 months of first dose of palliative chemotherapy. N = 840 for GEMNAB and gemcitabine for matched analyses.

## Discussion

In this large, population-based cohort study of patients with metastatic pancreas cancer treated with first-line palliative chemotherapy, several interesting findings have emerged. First, over time, as FOLFIRINOX and GEMNAB were funded, single-agent gemcitabine use became less common. When all 3 agents were available, single-agent gemcitabine use was almost exclusively restricted to older patients with higher comorbidity scores and lower performance status. Second, in the period in which only single-agent gemcitabine and FOLFIRINOX were funded, uptake of FOLFIRINOX was slow, and 50% of patients were treated with single-agent gemcitabine. In contrast, when GEMNAB was funded and all 3 agents were available, there was an immediate and sustained decline in the use of gemcitabine with stable rates of FOLFIRINOX use. This suggests that there exists a population of patients that oncologists determined were well enough to receive a more aggressive regimen than single-agent gemcitabine, but not well enough to receive FOLFIRINOX. This is supported by the observation that when both FOLFIRINOX and GEMNAB were funded for use, FOLFIRINOX use was not supplanted by GEMNAB, and that FOLFIRINOX was more commonly selected for use in younger patients with better ECOG performance status. In adjusted, propensity-weighted and matched survival analyses, patients treated with GEMNAB had better survival than patients treated with single-agent gemcitabine. This suggests that there may be a distinct population of patients who derive a survival benefit from GEMNAB that was not already being realized through availability of FOLFIRINOX. Taken together, these findings highlight that, over time, drug funding decisions were associated with increased uptake of new treatment options over time and improved survival for patients with advanced pancreatic cancer and that FOLFIRINOX and GEMNAB were associated with survival benefits in distinct patient populations.

This study adds to a growing body of literature evaluating the practice patterns and outcomes of patients with pancreatic cancer in routine clinical practice. Mavros et al^18^ conducted a population-based analysis of all incident cases of advanced pancreatic cancer in Ontario, Canada (2005-2016) to evaluate rates of medical oncology referral and systemic therapy receipt. Among 10 881 patients diagnosed with advanced pancreatic cancer, only 7062 (65%) had a consultation with a medical oncologist and only 4144 (38%) received systemic therapy. Among patients who saw a medical oncologist, older age (age ≥80 years vs ≤60 years: adjusted risk ratio [aRR], 0.48; 95% CI, 0.48-0.57) and higher comorbidity score (Aggregated Diagnosis Group ≥10 vs <10: aRR, 0.92; 95% CI, 0.84–1.02) were associated with lower chance of chemotherapy treatment. Conversely, patients with a more recent diagnosis were more likely to receive chemotherapy (2005-2010 vs 2011-2016: aRR, 1.20; 95% CI, 1.09-1.31). Similarly, O’Reilly et al^19^ used the Flatiron databases to evaluate treatment patterns and survival outcomes among patients with metastatic pancreatic cancer (2014-2019) in the United States. Among 7666 patients, 5687 (74%) received at least one line of systemic therapy.^19^ In a propensity-score matched analysis comparing patients who received systemic therapy against those who did not, treated patients had a significantly improved median overall survival of 8.1 vs 2.6 months (HR, 0.41; 95% CI, 0.38-0.45). It is of important note that performance status information was not available for 77% of patients.^19^

The present study builds upon these previous efforts and provides real-world evidence of improving patient outcomes over time for patients treated with systemic therapy for advanced pancreatic cancer. Moreover, the present study provides evidence that the uptake of FOLFIRINOX and GEMNAB appear to be associated with benefit for distinct populations of patients. To determine if GEMNAB was associated with a population-level survival benefit that was not already being realized through the availability of FOLFIRINOX, we compared the survival outcomes of patients with GEMNAB in the most recent period with those patients treated with gemcitabine in the preceding period when only gemcitabine and FOLFIRINOX were funded. We hypothesized that during the time when only gemcitabine and FOLFIRINOX were available, given the better survival outcomes associated with FOLFIRINOX, a key reason why the clinician would choose to treat a patient with gemcitabine was because they were concerned the patient did not have a sufficient performance status for FOLFIRINOX, yet could have received GEMNAB if this treatment was available at the time. In these analyses, GEMNAB was associated with survival improvements over gemcitabine in adjusted, weighted and matched analyses. These findings are of particular relevance to health system payers, and demonstrate the importance of real-world evidence to inform drug-funding decisions.^20^

### Limitations

The results of our study should be interpreted within the context of the methodological limitations. First, our current analysis is limited to all incident cases of advanced pancreas cancer that were treated with first-line chemotherapy. Second, we do not have access to information on the use of second-line chemotherapies, although it is of note that there are no government-funded second-line chemotherapy options in Ontario, Canada, other than single-agent gemcitabine after FOLFIRINOX. Third, our study cohort predates the approval of funding for FOLFIRINOX and gemcitabine and capecitabine in the adjuvant setting.^21^ Fourth, although our survival analyses used propensity score methods aimed to minimize the imbalance of confounders between treatment groups on outcomes, important difference may remain between treatment groups, and selection bias may continue to influence the observed treatment effect estimates. Finally, information on quality of life and cost-effectiveness are additional essential considerations to inform the selection of, and funding for, pancreatic cancer therapies.

## Conclusions

In this population-based study of patients with advanced pancreatic cancer receiving first-line palliative chemotherapy within a universal health care system, we observe that drug funding decisions were associated with increased uptake of new treatment options over time. Both FOLFIRINOX and GEMNAB were associated with improvements in survival for distinct patient populations. These findings suggest that drug-funding reimbursement decisions are associated improvements in survival for patients with advanced pancreatic cancer.

## Notes

Supplement. **eTable 1.** Description of Administrative Healthcare Database and the Information They Provided **eTable 2.** Baseline Characteristics After Inverse Probability of Treatment Weighting Applied Among Patients Treated With FOLFIRINOX and Gemcitabine + Nabpaclitaxel in Period 3 **eTable 3.** Adjusted and Inverse Probability of Treatment Weighted Hazard Ratios For Overall Survival By Treatment Regimen and Baseline Characteristics **eTable 4.** Baseline Characteristics After IPTW Applied Among Patients Treated With Gemcitabine in Period 2 (2011-2015) and Those Treated With Gemcitabine + Nab-Paclitaxel in Period 3 (2015-2018) **eFigure 1.** Kaplan-Meier Analysis of Overall Survival for Patients with Advanced Pancreas Cancer Receiving Treatment with First-line Chemotherapy between 2008-2011, 2011-2015 and 2015-2018 **eFigure 2.** Kaplan-Meier Plot for Overall Survival for Patients With Advanced Pancreatic Cancer Receiving Treatment With First-line Chemotherapy **eFigure 3.** Unweighted Kaplan-Meier Analysis of Overall Survival in Patients With Treated With Gemcitabine + Nab-Paclitaxel and FOLFIRINOX **eFigure 4.** IPT Weighted Kaplan-Meier Analysis of Overall Survival in Patients With Treated With Gemcitabine + Nab-Paclitaxel and FOLFIRINOX
