# Supplementary material for: The Association of Drug-Funding Reimbursement With Survival Outcomes and Use of New Systemic Therapies Among Patients With Advanced Pancreatic Cancer
Source: JAMA Netw Open. 2021 Nov 15;4(11):e2133388. doi: 10.1001/jamanetworkopen.2021.33388 (PMC8593760; doi:10.1001/jamanetworkopen.2021.33388)
Supplement: Supplement. — eTable 1. Description of Administrative Healthcare Database and the Information They Provided eTable 2. Baseline Characteristics After Inverse Probability of Treatment Weighting Applied Among Patients Treated With FOLFIRINOX and Gemcitabine + Nabpaclitaxel in Period 3 eTable 3. Adjusted and Inverse Probability of Treatment Weighted Hazard Ratios For Overall Survival By Treatment Regimen and Baseline Characteristics eTable 4. Baseline Characteristics After IPTW Applied Among Patients Treated With Gemcitabine in Period 2 (2011-2015) and Those Treated With Gemcitabine + Nab-Paclitaxel in Period 3 (2015-2018) eFigure 1. Kaplan-Meier Analysis of Overall Survival for Patients with Advanced Pancreas Cancer Receiving Treatment with First-line Chemotherapy between 2008-2011, 2011-2015 and 2015-2018 eFigure 2. Kaplan-Meier Plot for Overall Survival for Patients With Advanced Pancreatic Cancer Receiving Treatment With First-line Chemotherapy eFigure 3. Unweighted Kaplan-Meier Analysis of Overall Survival in Patients With Treated With Gemcitabine + Nab-Paclitaxel and FOLFIRINOX eFigure 4. IPT Weighted Kaplan-Meier Analysis of Overall Survival in Patients With Treated With Gemcitabine + Nab-Paclitaxel and FOLFIRINOX [file jamanetwopen-e2133388-s001.pdf]

Supplemental Online Content

Raphael MJ, Raskin W, Habbous S, et al. The association of drug-funding reimbursement with survival outcomes and use of new systemic therapies among patients with advanced pancreatic cancer. *JAMA Netw Open.* 2021;4(11):e2133388. doi:10.1001/jamanetworkopen.2021.33388

eTable 1. Description of Administrative Healthcare Database and the Information They Provided

eTable 2. Baseline Characteristics After Inverse Probability of Treatment Weighting Applied Among Patients Treated With FOLFIRINOX and Gemcitabine + Nabpaclitaxel in Period 3

eTable 3. Adjusted and Inverse Probability of Treatment Weighted Hazard Ratios For Overall Survival By Treatment Regimen and Baseline Characteristics

eTable 4. Baseline Characteristics After IPTW Applied Among Patients Treated With Gemcitabine in Period 2 (2011-2015) and Those Treated With Gemcitabine + Nab-Paclitaxel in Period 3 (2015-2018)

eFigure 1. Kaplan-Meier Analysis of Overall Survival for Patients with Advanced Pancreas Cancer Receiving Treatment with First-line Chemotherapy between 2008-2011, 2011-2015 and 2015-2018

eFigure 2. Kaplan-Meier Plot for Overall Survival for Patients With Advanced Pancreatic Cancer Receiving Treatment With First-line Chemotherapy

eFigure 3. Unweighted Kaplan-Meier Analysis of Overall Survival in Patients With Treated With Gemcitabine + Nab-Paclitaxel and FOLFIRINOX

eFigure 4. IPT Weighted Kaplan-Meier Analysis of Overall Survival in Patients With Treated With Gemcitabine + Nab-Paclitaxel and FOLFIRINOX

This supplemental material has been provided by the authors to give readers additional information about their work.

© 2021 Raphael MJ et al. *JAMA Network Open.*

eTable 1. Description of administrative healthcare database and the information they provided

Data source	Variables
New Drug Funding Program	Prior history of adjuvant gemcitabine<br>Cohort classification as gemcitabine monotherapy, FOLFIRINOX, or GEMNAB<br>Disease extent (uLAPC vs mPC)<br>ECOG<br>Postal code at enrollment<br>Physician name
Discharge Abstract Database (CIHI-DAD)	Charlson comorbidity score<br>Prior resection<br>History of hospital admission
National Ambulatory Care Reporting System (CIHI-NACRS)	Charlson comorbidity score<br>Prior resection<br>History of emergency department use
Activity Level Reporting	Prior history of radiation
Registered Persons Database	Vital statistics for overall survival<br>Demographics
Ontario Cancer Registry	Vital statistics for overall survival<br>Diagnosis date<br>Demographics<br>Tumor topography<br>Postal code at diagnosis
Postal Code Conversion File	Neighbourhood income quintile<br>Neighbourhood immigrant density<br>Rurality (urban vs. rural)
Corporate Provider Database	Physician specialty
College of Physicians and Surgeons of Ontario (CPSO) https://www.cpso.on.ca/	Physician specialty<br>International training<br>Years since medical degree obtained

ECOG = Eastern Cooperative Oncology Group; uLAPC = Unresectable and locally advanced pancreatic; cancer; mPC = Metastatic pancreas cancer; ED = Emergency department

© 2021 Raphael MJ et al. *JAMA Network Open.*

eTable 2. Baseline characteristics after inverse probability of treatment weighting applied among patients treated with FOLFIRINOX and Gemcitabine + Nabpaclitaxel in period 3

	FOLFIRINOX	Gemcitabine and Nab-paclitaxel	Weighted Standardized Difference
Age treatment started (mean +/- standard deviation)	65.64 +/- 12.51	64.94+/-15.55	0.0495
Female (%)	42.42	43.9907385	0.0318
Urban Dwelling (%)	87.32	88.0190048	0.0212
Income quintile (%)			
Lowest	14.18	13.26	0.0269
Mid-low	19.49	18.81	0.0172
Middle	21.03	21.57	0.0133
Mid-high	22.71	21.88	0.02
Highest	22.59	24.48	0.0446
Immigrant density (%)			
Most dense	13.21	12.47	0.0224
Mid-dense	23.73	23.15	0.0138
Least dense	63.05	64.38	0.0277
Charlson comorbidity			
1	17.81	17.02	0.0206
2+	5.86	6.42	0.0231
ECOG 1+ (%)	71.19	69.93	0.0277
mPC (%)	70.03	69.29	0.0162
Prior adjuvant gemcitabine (%)	9.85	9.43	0.0143
Prior adjuvant radiation (%)	7.76	7.29	0.0179
Prior pancreas resection (%)	14.74	14.43	0.0088
Baseline ED Visit* (%)			
1 visit	14.48	14.16	0.0092
2+ visits	31.96	32.10	0.0031
Baseline Hospital Admission* (%)			
1 admission	29.89	28.83	0.0233
2+ admissions	19.53	20.40	0.0219

ECOG = Eastern Cooperative Oncology Group; uLAPC = Unresectable and locally advanced pancreatic; cancer; mPC = Metastatic pancreas cancer; ED = Emergency department

*Baseline = Within 6 months before first dose of palliative chemotherapy.

© 2021 Raphael MJ et al. *JAMA Network Open.*

eTable 3. Adjusted and inverse probability of treatment weighted hazard ratios for overall survival by treatment regimen and baseline characteristics

	**Adjusted Cox Proportional Hazard Model***	**Weighted Adjusted Cox Proportional Hazard Model***
FOLFIRINOX vs. GEMNAB	0.78 (0.70-0.87)	0.77 (0.73-0.83)
Age treatment started (per year)	1.01 (1.00-1.01)	1.00 (1.00-1.01)
Female vs. Male	0.94 (0.86-1.03)	0.93 (0.87-0.99)
Urban vs. Rural Dwelling	1.03 (0.89-1.20)	1.09 (0.99-1.21)
Income quintile		
Lowest	Ref	Ref
Mid-low	0.93 (0.80-1.10)	1.10 (0.98-1.23)
Middle	0.83 (0.71-0.98)	0.93 (0.83-1.05)
Mid-high	0.84 (0.72-0.99)	0.97 (0.87-1.09)
Highest	0.92 (0.78-1.08)	1.03 (0.92-1.16)
Immigrant Density		
Most	Ref	Ref
Mid	1.02 (0.87-1.19)	0.99 (0.88-1.11)
Least	1.13 (0.98-1.31)	1.09 (0.98-1.21
Charlson comorbidity		
0	Ref	Ref
1	1.07 (0.94-1.20)	1.15 (1.06-1.26)
2+	1.22 (1.01-1.48)	1.27 (1.11-1.45)
ECOG 0 vs. ECOG 1+	0.65 (0.58-0.73)	0.65 (0.60-0.70)
uLAPC vs. mPC	0.64 (0.57-0.71)	0.67 (0.62-0.72)
Prior adjuvant gemcitabine	0.93 (0.77-1.13)	1.04 (0.91-1.19)
Prior radiation	1.15 (0.96-1.37)	1.25 (1.11-1.42)
Prior resection	0.94 (0.80-1.11)	0.80 (0.71-0.90)
Baseline ED Visit**		
0	Ref	Ref
1	1.36 (1.01-1.83)	1.11 (0.91-1.36)
2+	1.41 (1.05-1.89)	1.19 (0.98-1.46)
Baseline hospital admission**		
0	Ref	Ref
1	0.89 (0.67-1.18)	1.07 (0.89-1.30)
2+	0.89 (0.65-1.22)	0.98 (0.79-1.22)

GEMNAB = Gemcitabine + Nab=paclitaxel; ED = Emergency Department.

*Adjusted for all variables in the table using Cox proportional hazard regression modelling.

**Baseline = Within 6 months before first dose of palliative chemotherapy.

© 2021 Raphael MJ et al. *JAMA Network Open.*

eTable 4. Baseline characteristics after IPTW applied among patients treated with gemcitabine in period 2 (2011-2015) and those treated with gemcitabine + nab-paclitaxel in period 3 (2015-2018)

	Gemcitabine and Nab-paclitaxel (N=1107)	Gemcitabine (N=958)	Weighted Standardized Difference
Age treatment started (mean +/- standard deviation)	69.97 +/- 13.70	70.00 +/- 11.89	0.0021
Female (%)	43.42	42.65	0.0157
Urban Dwelling (%)	88.55	88.94	0.0125
Income quintile (%)			
Lowest	15.89	16.32	0.0116
Mid-low	18.53	18.23	0.0079
Middle	21.15	21.00	0.0035
Mid-high	22.53	21.87	0.0158
Highest	21.90	22.58	0.0162
Immigrant density (%)			
Most dense	16.37	16.59	0.0061
Mid-dense	25.03	25.33	0.0068
Least dense	58.60	58.08	0.0106
Charlson comorbidity (%)			
1	19.53	19.72	0.0049
2+	6.59	6.52	0.0027
Prior adjuvant gemcitabine (%)	4.45	3.69	0.0384
Prior adjuvant radiation (%)	10.45	10.55	0.003
Prior pancreas resection (%)	14.90	14.51	0.011
Baseline ED Visit* (%)			
1 visit	17.59	17.67	0.0021
2+ visits	34.96	34.89	0.0014
Baseline Hospital Admission* (%)			
1 admission	33.26	33.03	0.005
2+ admissions	23.59	23.97	0.009

Data expressed as percentages unless otherwise stated.

ED = Emergency department

*Baseline = Within 6 months before first dose of palliative chemotherapy.

© 2021 Raphael MJ et al. *JAMA Network Open.*

eFigure 1. Kaplan-Meier Analysis of Overall Survival for Patients with Advanced Pancreas Cancer Receiving Treatment with First-Line Chemotherapy between 2008-2011, 2011-2015 and 2015-2018.

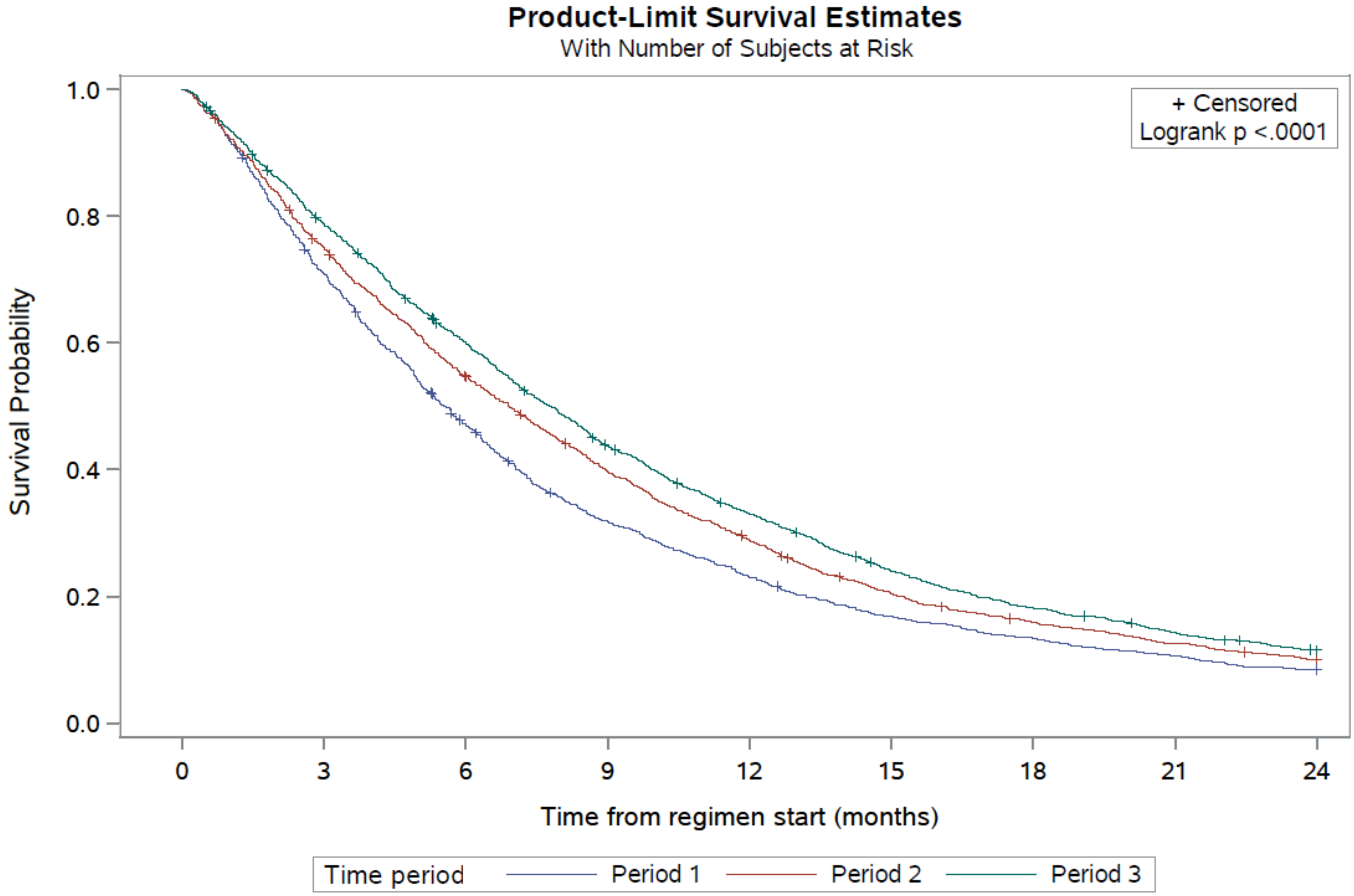

Period 1	1231	871	576	386	278	203	162	128	102
Period 2	1887	1415	1030	747	539	379	293	232	185
Period 3	2347	1843	1404	1016	765	554	419	327	261

© 2021 Raphael MJ et al. *JAMA Network Open.*

eFigure 2. Kaplan-Meier plot for overall survival for patients with advanced pancreatic cancer receiving treatment with first-line chemotherapy. Follow-up started from the time the patient started chemotherapy with gemcitabine (Gem), gemcitabine with nab-paclitaxel (Gnp), or FOLFIRINOX (FFX) stratified by time period (a) or regimen and time period (b). Period 1 (P1) corresponds to November 7, 2008 to November 6, 2011 (only gemcitabine was funded). Period 2 corresponds to November 7, 2011 to April 14, 2015 (only gemcitabine and FOLFIRINOX were funded). Period 3 corresponds to April 15, 2015 to December 31, 2018 (gemcitabine, FOLFIRINOX, and Gnp were funded).

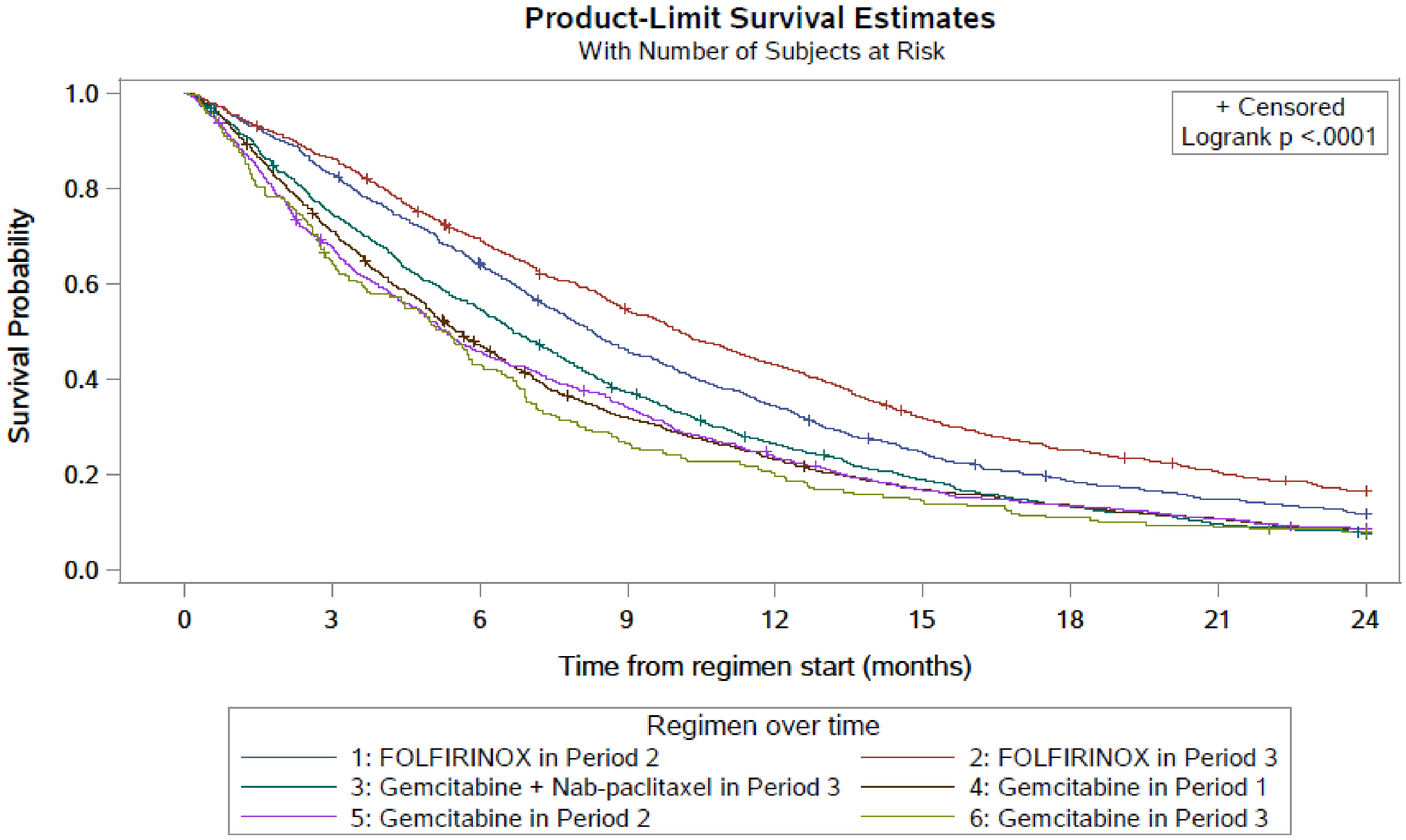

1	929	771	595	424	317	223	167	133	106
2	1034	890	714	555	439	322	254	205	165
3	1107	822	602	407	286	203	143	104	81
4	1231	871	576	386	278	203	162	128	102
5	958	644	435	323	222	156	126	99	79
6	206	131	88	54	40	29	22	18	15

© 2021 Raphael MJ et al. *JAMA Network Open.*

eFigure 3. Unweighted Kaplan-Meier analysis of overall survival in patients with treated with Gemcitabine + Nab-paclitaxel and FOLFIRINOX

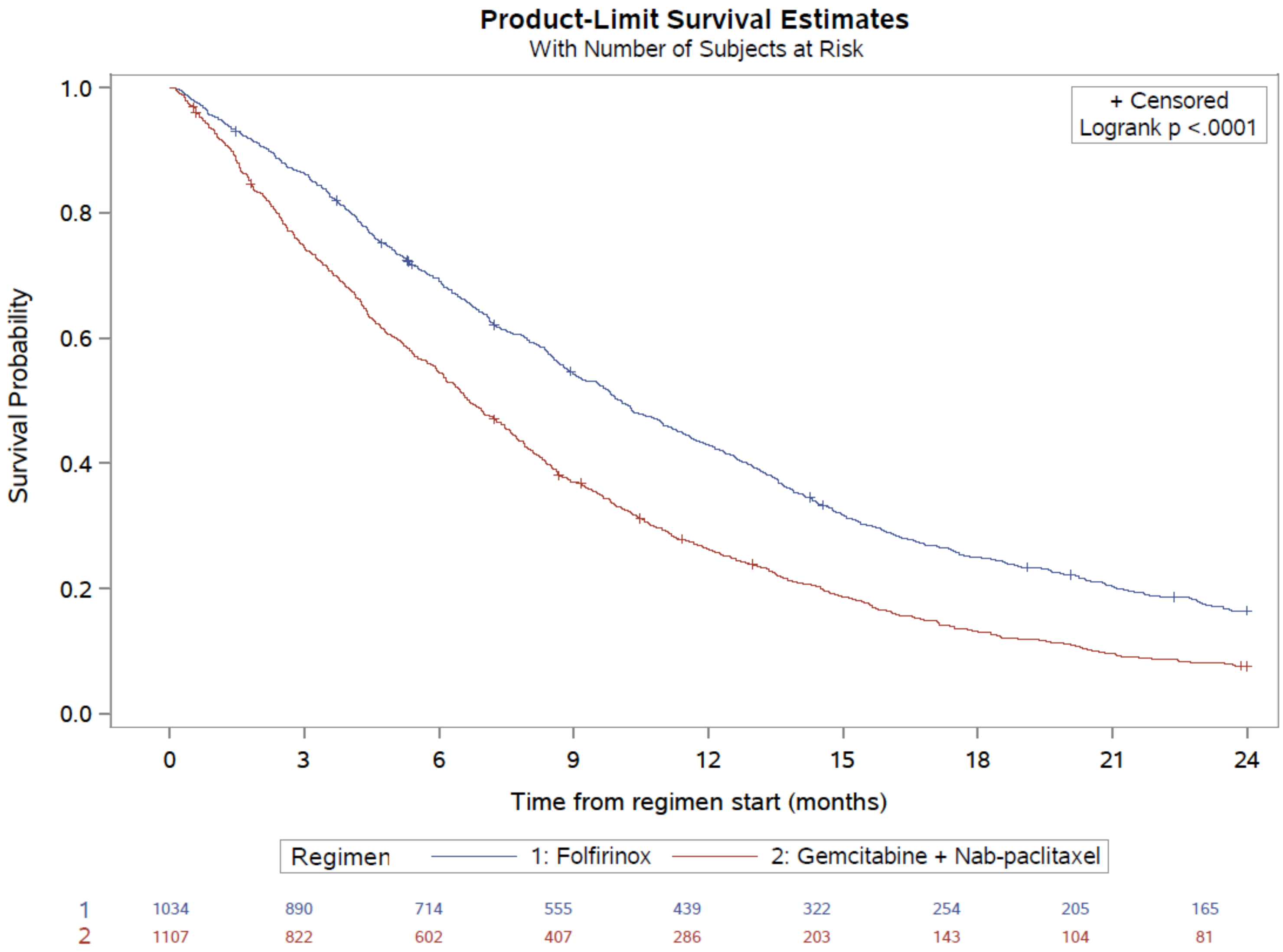

© 2021 Raphael MJ et al. *JAMA Network Open.*

eFigure 4. IPT weighted Kaplan-Meier analysis of overall survival in patients with treated with gemcitabine + nab-paclitaxel and FOLFIRINOX

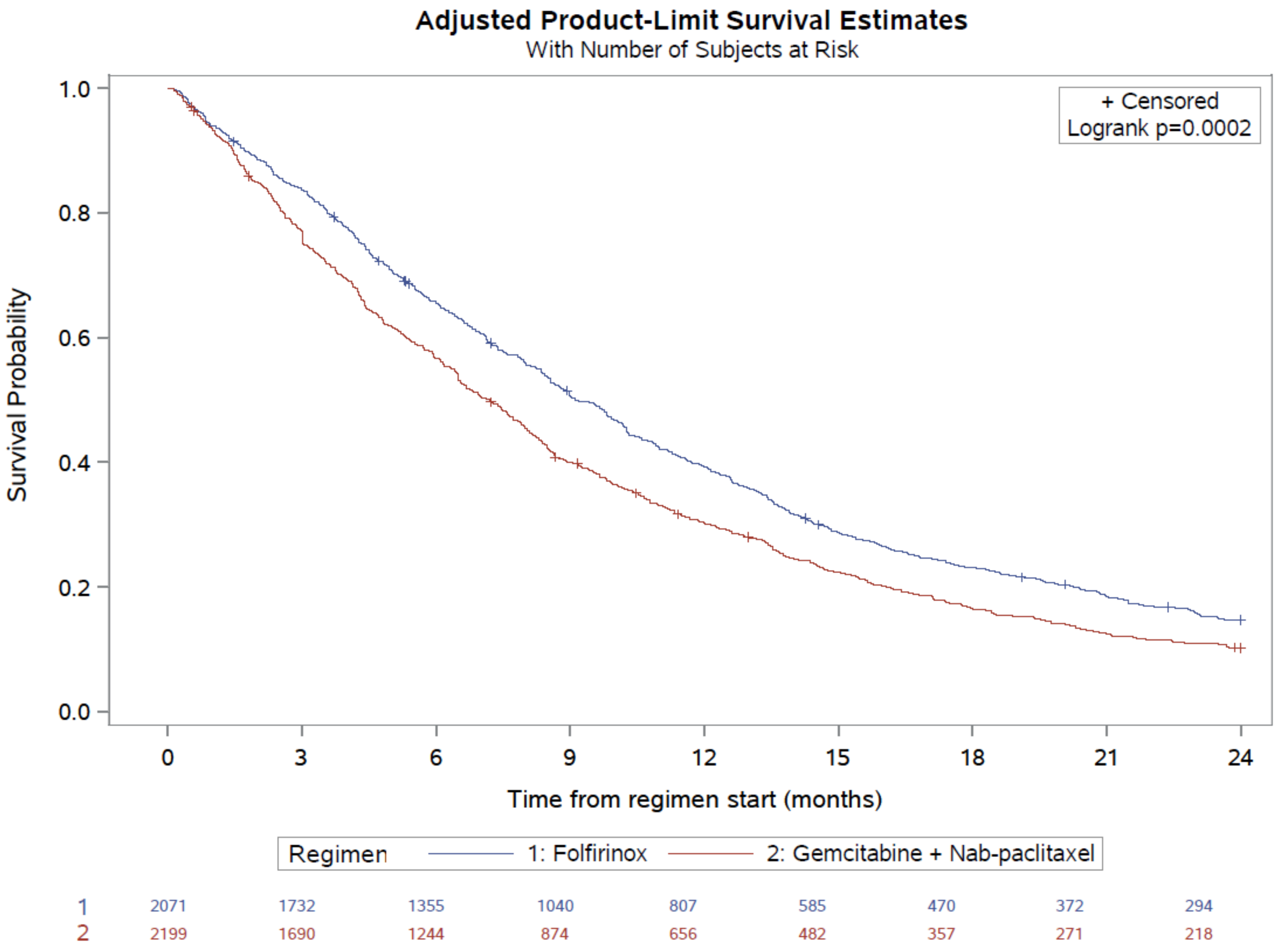

© 2021 Raphael MJ et al. *JAMA Network Open.*
